# Characterization of Micro-Crack Orientation in a Thin Plate Using Quasi-Static Component Generated by Incident Ultrasonic Lamb Waves

**DOI:** 10.3390/s25010222

**Published:** 2025-01-02

**Authors:** Liang Zhao, Jun Zhou, Weifeng Yuan, Bin Gu, Mingxi Deng, Caibin Xu, Xiangyan Ding, Zhengpan Qi, Jishuo Wang, Qin Ying

**Affiliations:** 1 Key Laboratory of Testing Technology for Manufacturing Process MOE, Southwest University of Science and Technology, Mianyang 621010, China; 2 Shock and Vibration of Engineering Materials and Structures Key Laboratory of Sichuan Province, Southwest University of Science and Technology, Mianyang 621010, China; 3 School of Mechanical Engineering, Hebei University of Technology, Tianjin 300401, China; 4 College of Aerospace Engineering, Chongqing University, Chongqing 400044, China

**Keywords:** micro-crack, orientation, quasi-static component, Lamb wave, bilinear stress–strain model

## Abstract

The directivity of the quasi-static component (QSC) is quantitatively investigated for evaluating the orientation of a micro-crack buried in a thin solid plate using the numerical simulation method. Based on the bilinear stress–strain constitutive model, a three-dimensional (3D) finite element model (FEM) is built for investigating the nonlinear interaction between primary Lamb waves and the micro-crack. When the primary Lamb waves at A0 mode impinge on the micro-crack, under the modulation of the contact acoustic nonlinearity (CAN), the micro-crack itself will induce QSC. The amplitude of the QSC generated can be used for directly charactering the micro-crack orientation. The finite element simulation results show that the directivity of the QSC radiated by the micro-crack is closely related to the orientation of the micro-crack, allowing for the characterization of micro-crack orientation without the need for baseline signals. The results indicate that the directionality of the QSC can be used for characterizing the orientation of the micro-crack. The amplitude of the QSC is affected by the contact area between two surfaces of the micro-crack. It is demonstrated that the proposed method is a feasible means for the characterization of micro-crack orientation.

## 1. Introduction

Micro-crack is a potential cause of material failure and fracture, and its direction affects the path and speed of crack propagation, affecting the material’s durability and safety. Sheet metal structures are widely used in aerospace, architecture, and other fields because of their good plasticity and flexural properties. These metal sheets will be affected by various factors in service, such as stress concentration, acid–base corrosion, fatigue and high temperature, etc., which are liable to cause dislocation and slip within them under the combined action of these factors, resulting in the formation of closed cracks and pseudo-bonding [1,2]. Studies have shown that the initiation and expansion stages of micro-crack account for 70% to 90% of the total fatigue life of parts [3,4], and the extension direction of micro-crack has a significant impact on the remaining life of mechanical parts [5]. Therefore, it is significant for material safety in service to characterize the micro-crack direction buried in thin sheet metal structures.

Ultrasonic wave technology has been widely used in the nondestructive testing field due to its high detection efficiency and sensitivity to early damage in a thin solid plate. It is mainly based on the principles of crack reflection [4], diffraction [6], and transmission [7], which can detect macro-crack [8], but nothing can be done to detect micro-crack. In recent years, it has been found that the interaction between ultrasonic waves and early damage will induce strong nonlinear responses, such as second harmonics [9,10,11,12,13], quasi-static components (QSCs) [14,15,16,17,18,19,20,21,22,23,24], and mixing harmonics [25,26,27]. Therefore, nonlinear ultrasonic technology has attracted more and more attention.

The generation mechanism of nonlinear acoustic signals generated by the interaction between the ultrasonic waves and micro-crack has been widely investigated in recent years. Solodov et al. [28] built a contact acoustic nonlinearity (CAN) phenomenological model for explaining the nonlinear acoustic phenomena observed for the interaction of ultrasonic waves with simulated and realistic nonbonded contact interfaces (cracked defects) in solids. Broda et al. [29] reviewed the interaction mechanism between micro-crack and ultrasonic waves. The generation mechanism and damage evaluation of the second harmonic of ultrasonic Lamb wave [30] and mixing harmonic have been very mature [26,31]. Yelve et al. [32] used second harmonics to detect cracks buried at different depths. Zhang et al. [33] combined pulse inverse technology with continuous wavelet transform for detecting cracks in complex structures. Liu et al. [34] found that the acoustic nonlinear parameter monotonically increases with the increase in micro-crack density, region size, resonant wave frequency, and the surface friction coefficient of micro-crack. Nowadays, the nonlinear ultrasonic response modulated by micro-crack has been widely studied. Wang et al. [35] quantitatively studied the orientation characteristics of the micro-crack by using the directivity of the second harmonic radiated by the secondary sound source caused by the nonlinear interaction between the incident ultrasonic shear wave and the micro-crack. Lv et al. [36] proposed a non-collinear shear wave mixing technique for evaluating fatigue crack orientation. Blanloeuil et al. [37] extended the numerical solution analytically in the far field based on the frequency domain near-field to far-field transformation technology. They obtained the directivity of the numerical solution. Zhang et al. [38] used the scattering coefficient matrix of the crack defect to acquire its size, shape, and orientation.

Nonlinear acoustic characteristic signals, such as the second harmonic [11,12,13], mixing harmonic [26,27], etc., are usually used for quantitatively evaluating micro-crack. It has been widely investigated since the QSC can also be simultaneously generated with other harmonics in a thin plate. Hu et al. [14,21] theoretically proved the existence of the QSC of ultrasonic Lamb waves in weak nonlinear materials and quantitatively evaluated the cumulative plastic damage based on the QSC. Wan et al. [39] analyzed the propagation characteristics of the QSC of ultrasonic Lamb waves in thin plates from theoretical and simulation perspectives. Chen et al. [17] studied the generation mechanism of the QSC of ultrasonic Lamb waves in laminates from a theoretical and numerical simulation perspective. Gao et al. [18] verified the propagation characteristics of the QSC theoretically and experimentally. When the group velocity of ultrasonic primary Lamb wave matches that of the QSC, the amplitude of the QSC will linearly accumulate with propagation distance. Sun et al. [19] showed that, compared to the second harmonic method, the detection technology based on the QSC is more efficient and has a strong characterization of the early damage.

The QSC is highly sensitive to early damage and is often used to detect material microstructure damage and early damage, and even tiny defects can produce significant additional nonlinearity. Compared to the second harmonics, the QSC overcomes the limitations of traditional nonlinear Lamb waves because it does not need phase velocity matching and features low attenuation. Since the micro-crack orientation affects the characteristic coefficient, and few works [35,36] have focused on the micro-crack orientation, it is necessary to study the relationship between the direction of micro-cracks and the QSC.

To solve the problem, a finite element model (FEM) for simulating the ultrasonic sound field of the QSC is built, and the quantitative relationship between the orientation of the micro-crack and QSC directivity is established. The remainder of this paper is structured as follows: in Section 2, the theory of CAN, the bilinear stress–strain model, the propagating characteristic of the QSC, and the extraction process of the QSC are given. The physical mechanism of the nonlinear interaction between incident primary Lamb waves (PLWs) and the micro-crack is presented, and an FEM based on CAN is established. The propagation characteristic between PLWs and the QSC under group velocity mismatch is analyzed. In Section 3, the simulation results are presented to discuss the relationship between QSC directivity and micro-crack orientation. The conclusions are drawn in Section 4.

## 2. Theoretical Fundamental

### 2.1. Bilinear Stress–Strain Constitutive Law

**Figure 1:**
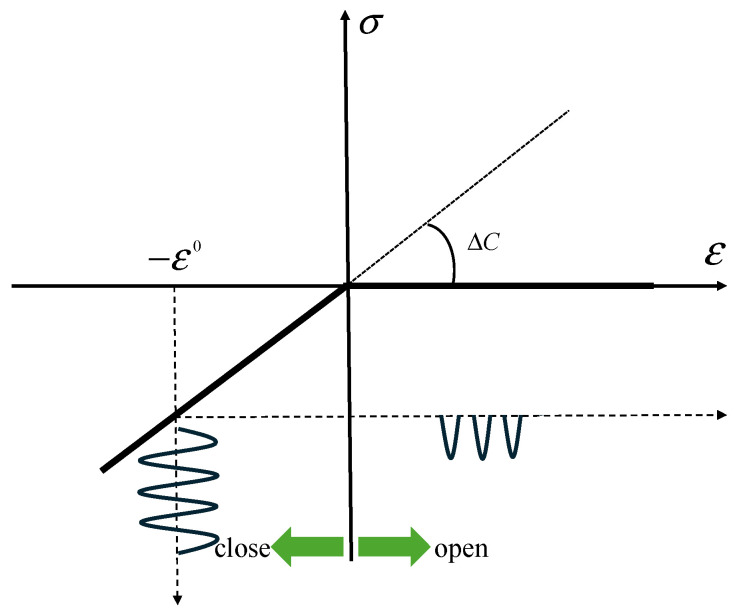
The bilinear constitutive relationship between stress and strain of the micro-crack.

The bilinear stress–strain law is widely used for describing the mechanical behavior of the micro-crack. The law is defined as the CAN effect induced by the nonlinear interaction between the ultrasonic waves and micro-crack [40]. The fact of the CAN is that the region around the micro-crack lacks stiffness symmetry. When the compressive phase of the ultrasonic wave comes to the area, its stiffness remains unchanged, as shown in Figure 1, where the negative semi-axis shows that the micro-crack remains closed. The ultrasonic wave now penetrates the micro-crack, propagating through an intact continuous medium. In another case, when the tensile phase of the ultrasonic wave propagates to the micro-crack region, the stiffness of the region is lost, as shown in Figure 1. Then, the waveform is distorted, inducing the new QSC and harmonic components. The combined actions of those two phases give rise to the nonlinear interaction between the ultrasonic wave and the examined micro-crack. A bilinear stress–strain constitutive law can simulate such a “bi-modular” area [28]:

(1) $$\sigma =C\left[1-H\left(\varepsilon -\varepsilon^{0}\right)\left(\Delta C/C\right)\right]\varepsilon$$

where H(*) is the Heaviside unit step function; $\varepsilon^{0}$ is the initial static contact strain, which defines the so-called “operating point”; and $\Delta C=\left[C-{\left(d\sigma /d\varepsilon \right)}_{\varepsilon >0}\right]$ is the difference in the elasticity modular between compressive and tensile phases.

### 2.2. Analysis of QSC Propagating Characteristic Under Mismatch Condition of Group Velocity

Detecting and evaluating the micro-crack is always a challenging problem using nonlinear ultrasonic technology because nonlinear ultrasonic component (the second harmonic, mixing frequency harmonic, etc.) energy is two orders of magnitude smaller than that of the primary ultrasonic waves. Improving nonlinear ultrasonic component energy is always a hot topic of research. The emergence of the QSC provides a new approach for solving the problem. Previous works [18] have shown that when the group velocity is matched, the generated QSC’s duration is the same as that of the incident primary Lamb wave. The QSC generated will be the superposition along the path propagation by PLWs. However, separating the main contribution from the material nonlinearity to the micro-crack is difficult. Group velocity mismatching between PLWs and the QSC is a better choice to weaken the disturbance from the material nonlinearity.

To better understand the generation and propagation characteristic of the QSC under group velocity mismatching in a thin plate, the perturbation approximation is utilized, and the detailed propagation process is analyzed. The traction tensors at zero frequency can be obtained on the upper and lower surfaces by employing a second-order perturbation. It is known that, within the second-order perturbation approximation [30,41], there is a distribution of the second-order bulk ($F_{V}$) and surface driving forces ($F_{S}$) along the propagation path of the PLW. The forces can be seen as a series of driving sources, which will generate secondary waves, including the QSC, the second harmonic, etc. The cumulative generation and propagation under different conditions of the second harmonic have been investigated. Still, the physical process of the generation and propagation of QSCs under group mismatching has not been studied in a single thin plate. In this section, the physical process of the QSC generation and propagation mechanism will be investigated. Because the cumulative of the generated QSC is not limited to the phase matching condition, its shape is only affected by the group velocity of the PLW. Here, the condition of group velocity mismatching for the generation and propagation mechanism of the generated QSC pulse is schematically illustrated.

**Figure 2:**
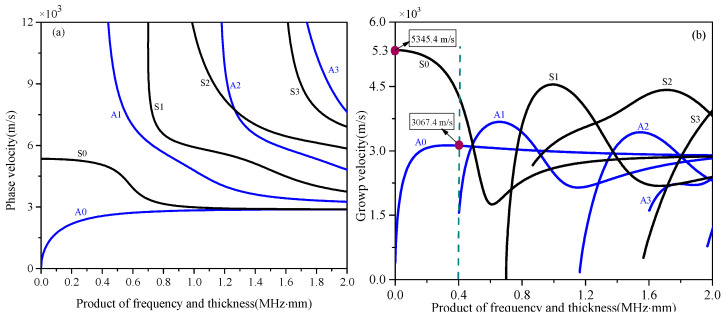
Dispersion curves of primary Lamb waves: (**a**) phase velocity; (**b**) group velocity.

The time-domain analysis of the QSC generation of PLW propagation under the condition of group velocity mismatching is considered. The disperse curves of group velocity in a solid thin plate is shown in Figure 2. As the $C_{g}^{\left[f\right]}<C_{g}^{\left[\mathrm{SC}\right]}$ ($C_{g}^{\left[f\right]},C_{g}^{\left[\mathrm{SC}\right]}$ are group velocities of the PLW and QSC, respectively) and $C_{g}^{\left[f\right]}>C_{g}^{\left[\mathrm{SC}\right]}$ are similar, as shown in Figure 2, the condition of $C_{g}^{\left[f\right]}<C_{g}^{\left[\mathrm{SC}\right]}$ is only analyzed. When the PLW waveform arrives at $z_{0}$ position, the time-of-flight of the generated QSC pulse (generated by the $F_{V}^{\left[\mathrm{SC}\right]}(z_{i},t)$ and $F_{S}^{\left[\mathrm{SC}\right]}(z_{i},t)$, which are the second-order bulk and surface driving force of the QSC) from the position *T* to *R* is $z/C_{g}^{\left[\mathrm{SC}\right]}$. In the same way, when the PLWs’ waveform arrives at an arbitrary position $z_{i}$, the time-of-flight of the generated QSC (generated by the $F_{V}^{\left[\mathrm{SC}\right]}(z_{i},t)$ and $F_{S}^{\left[\mathrm{SC}\right]}(z_{i},t)$) from $z_{i}$ to the position *R* is $\left(z-z_{i}\right)/C_{g}^{\left[\mathrm{SC}\right]}$, and the time-of-flight $z_{i}/C_{g}^{\left[f\right]}$ of the PLW waveform spends from $z_{0}$ to $z_{i}$. Due to the condition $C_{g}^{\left[f\right]}<C_{g}^{\left[\mathrm{SC}\right]}$, the QSC pulses generated at the position $z_{0}$ and $z_{i}$ will linearly travel to the position *R* and do not overlap completely. Clearly, the time-domain range of the QSC pulse generated at *Z*_0_ is from $z/C_{g}^{\left[\mathrm{SC}\right]}$ to $z/C_{g}^{\left[f\right]}+\tau$, and that of the QSC pulse generated at $z_{i}$ is from $z_{i}/C_{g}^{\left[f\right]}+\left(z-z_{i}\right)/C_{g}^{\left[\mathrm{SC}\right]}$ to $z_{i}/C_{g}^{\left[f\right]}+\left(z-z_{i}\right)/C_{g}^{\left[\mathrm{SC}\right]}+\tau$.

**Figure 3:**
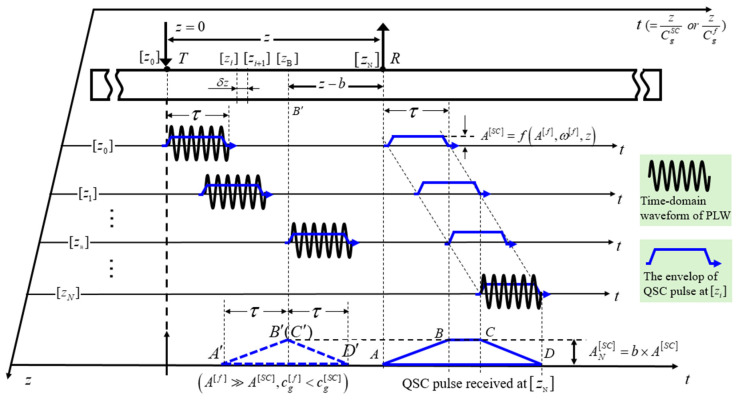
The physical process of generation of the time-domain QSC by primary Lamb wave under the group velocity mismatch in a thin plate.

Generally speaking, factors such as the duration of the PLW (namely τ), the difference between $C_{g}^{\left[f\right]}$ and $C_{g}^{\left[\mathrm{SC}\right]}$, and the propagation distance z determine the overlapping degree of those QSC pulses shown in Figure 3. To satisfy the purpose of the evaluation of micro-crack orientation, assume that the distance from *T* to *R* is far enough away (the distance is longer than $2\tau \cdot C_{g}^{\mathrm{SC}}$) that at one moment the tail of the QSC pulse generated at position $z_{0}$ overlaps with the head of that generated at $z_{B}$. Thus, the special position *B* can be determined by $z_{B}/c_{g}^{\left[f\right]}=z_{B}/c_{g}^{\left[f\right]}+\tau$, $z_{B}=\tau c_{g}^{\left[f\right]}c_{g}^{\left[SC\right]}/\left|c_{g}^{\left[f\right]}-c_{g}^{\left[SC\right]}\right|$.

Evidently, the time-domain length of the QSC ranges from $t=z_{B}/C_{g}^{\left[\mathrm{SC}\right]}$ to $t=z_{B}/C_{g}^{\left[f\right]}+\tau$ (namely $\bar{A^{\prime }B^{\prime }}$), as shown in Figure 3, and linearly increases with the traveling time of the PLW. Thus, the magnitude of the QSC pulse linearly increases with time in the range from $t=z_{B}/C_{g}^{\left[\mathrm{SC}\right]}$ to $t=z_{B}/C_{g}^{\left[f\right]}+\tau$ (namely the slope $\bar{AB}$). In the time-domain range from $t=z_{B}/C_{g}^{\left[f\right]}+\tau$ to $t=z_{N}/C_{g}^{\left[f\right]}+\tau$ (namely $\bar{BC}$), the overlapping degree of the QSC pulse received at position *R* is kept unchanged. The magnitude of the generated QSC pulse is kept constant in this time range. The maximum of the amplitude of the QSC received at the position *R* yields to $A_{N}^{\left[\mathrm{SC}\right]}=b\times A^{\left[\mathrm{SC}\right]}$, where *b* is the distance from the position *T* to the position $B^{\prime }$, and $A^{\left[\mathrm{SC}\right]}$ is the function of the amplitude, angle frequency, and propagation distance of the PLW.

The interest of investigating the propagation characteristic of the QSC is locating or imaging the micro-crack in a thin plate. Because the inherent nonlinearity of the materials cannot be eliminated, the effect of the generated QSC caused by the material should be as small as possible than that induced by the micro-crack. Therefore, the cumulative effect of the QSC pulse generated by the materials should not appear, so that the effect on the micro-crack orientation is as small as possible.

### 2.3. Nonlinear Interaction Between PLW and Micro-Crack

**Figure 4:**
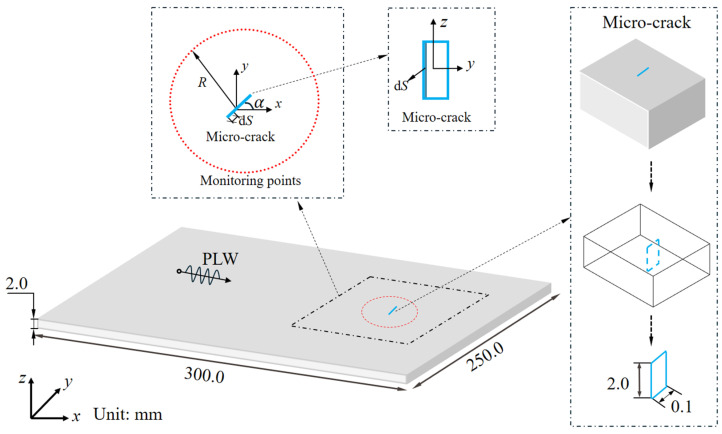
A 3D FEM of nonlinear interaction between an incident PLW and a micro-crack.

The nonlinear interaction mechanism between the PLW and the micro-crack is investigated. It is assumed that the angle of the micro-crack is α from the horizontal direction and is inconsistent with the vibration direction of the PLW (i.e., $0\leq \theta \leq 180^{\circ }$), as shown in Figure 4. Assuming that the wavelength of the PLW is λ, the micro-crack can be divided into *N* segments (the area of each segment is defined as dS, and the length and width of dS satisfy ≪λ). When the PLW propagates to the micro-crack region, each segment dS of the micro-crack interacts with it, and each segment dS of the micro-crack can be regarded as a secondary sound source radiating the QSC sound field. Then, the total QSC sound field can be considered to be the sum of each QSC sound field superposition. Since the secondary sound source is caused by the interaction between the micro-crack and PLW, the micro-crack is closely related to the incident PLW characteristics (such as amplitude $A_{inc}$, driving frequency $f_{inc}$, and phase $\varphi_{inc}$).

In addition, it is also related to the orientation angle of the micro-crack *α*. Thus, if the total QSC sound field radiated by d*S* is defined as $U_{0f}$, then it is a function of a series of parameters related to the incident PLW and the micro-crack (e.g., $A_{inc},f_{inc},\alpha$), i.e.,

(2) $$U_{0f}=f\left(A_{inc},f_{inc},\alpha \right)$$

The total sound field includes both the initial sound field of the incident PLW and the newly generated secondary sound field (including QSC, second harmonics, etc.). Without loss of generality, the initial sound field is defined as $U_{f}$, and the total sound field is defined as $U_{T}$. Therefore, the total sound field can be expressed as follows:

(3) $$U_{T}=U_{f}+U_{0f}$$

When the parameters (e.g., $A_{inc},f_{inc},\alpha$) in Equation (2) are given, the relevant characteristics (such as directivity) of the QSC sound field generated by the secondary sound source are closely related to the orientation of the micro-crack. Therefore, the directivity of the QSC radiated by the micro-crack can be used to evaluate the micro-crack orientation quantitatively.

In addition, due to the nonlinearity of the material itself, there are two nonlinearity sources in the numerical simulation: one is the nonlinearity of the material and the other is the nonlinearity caused by micro-cracks. To make QSC generated by micro-cracks dominate, the influence of material nonlinearity should be minimized. According to the propagation characteristic of the QSC in Section 2.2, when the group velocity mismatches, the QSC generated by the material nonlinearity will not continuously accumulate with the increase in the propagation distance, which will not affect the judgment of the QSC generated by the micro-crack. Therefore, to avoid the influence of material nonlinearity on the QSC, the low-frequency A0 mode Lamb wave, whose group velocity is very different from that of the QSC, is chosen here. In this case, the QSC caused by material nonlinearity can be ignored, and the micro-crack is regarded as the only nonlinear source.

### 2.4. Simulation Modeling

**Figure 5:**
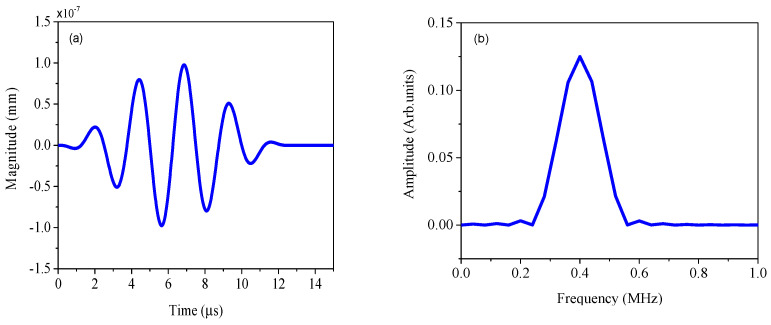
Excitation signals: (**a**) the time-domain diagram, (**b**) and the corresponding amplitude-frequency curve.

The material used in this simulation is an aluminum plate, and the FEM is built in ABAQUS 6.4.1, as shown in Figure 4. The length, width, and thickness of the finite element model are 300.0 mm, 250.0 mm, and 2.0 mm, respectively. The material parameters and group velocities of the PLW and QSC are shown in Table 1. As shown in Figure 4, the incident ultrasonic Lamb wave is a sinusoidal signal modulated by the Hanning window, with a center frequency of 0.4 MHz (the frequency $f_{inc}$ in Equation (2), a cycle number of 5, and an amplitude of $1\times 10^{-4}$ mm (the amplitude $A_{inc}$ in Equation (2). Figure 5a,b represent the time-domain signal and the corresponding amplitude–frequency curve of the incident PLW. The origin of the coordinate system is located in the geometrical center of FEM. In the FEM, angle α represents the orientation angle between the main direction of the micro-crack and the positive direction of the *x*-axis, where the length direction of the micro-crack is defined as its main direction. It is assumed that the center of the micro-crack with orientation angle α is at the center of *x* = 40 mm, *y* = 0 mm, and *z* = 0 mm. The micro-crack length is set to 0.1 mm and runs through the entire aluminum plate in the thickness direction. To facilitate the extraction of QSCs generated by the micro-crack, the monitoring point is set on the circumference of the upper surface, and its radius *R* is 20 mm.

**Figure 6:**
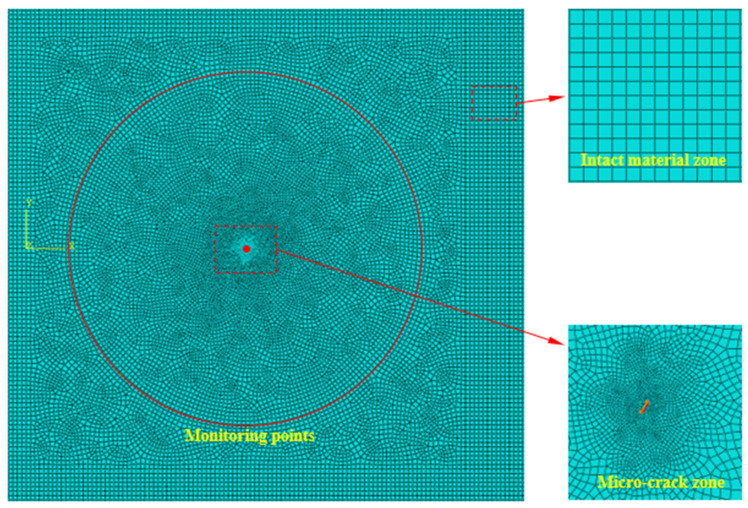
Meshes of local enlargement of the micro-crack zone and the remaining region of the model.

The element type is eight-node hexahedral elements (C3D8R), as shown in Figure 6. In the finite element (FE) simulation, the required spatial discrete length and time step must be calculated to make the results accurate enough. The mesh size and integral time step are obtained from the minimum wavelength and maximum frequency, respectively, and their values can be calculated according to the following equation:

(4) $$L_{e}\leq \frac{\lambda_{\min}}{10},\Delta t=\frac{1}{20f_{\max}}$$

where $\lambda_{\min}$ is the minimum wavelength of the PLW at a frequency of 0.4 MHz; $L_{e}$ is the maximum size of the meshes; Δt is the maximum time increment; and $f_{\max}$ is the maximum frequency.

Because the center frequency of the excitation signal is 0.4 MHz, the wavelength is 5.4 mm, the maximum mesh size is 0.54 mm, and the minimum time step is $1.25\times 10^{-7}\;s$. The mesh size is set to 0.5 mm, and the time step is selected $5.0\times 10^{-8}\;s$ to ensure the calculation accuracy and efficiency.

### 2.5. Extraction QSC Generated by Micro-Crack

When the PLW propagates from the excitation source to the micro-crack, the interaction between the PLW and micro-crack will produce nonlinear characteristic signals with micro-crack information, such as second harmonics, the QSC, etc. Since the amplitude of the nonlinear characteristic signal is two orders smaller than that of the PLW, how to extract the characteristic signal from the received signal is particularly important.

**Figure 7:**
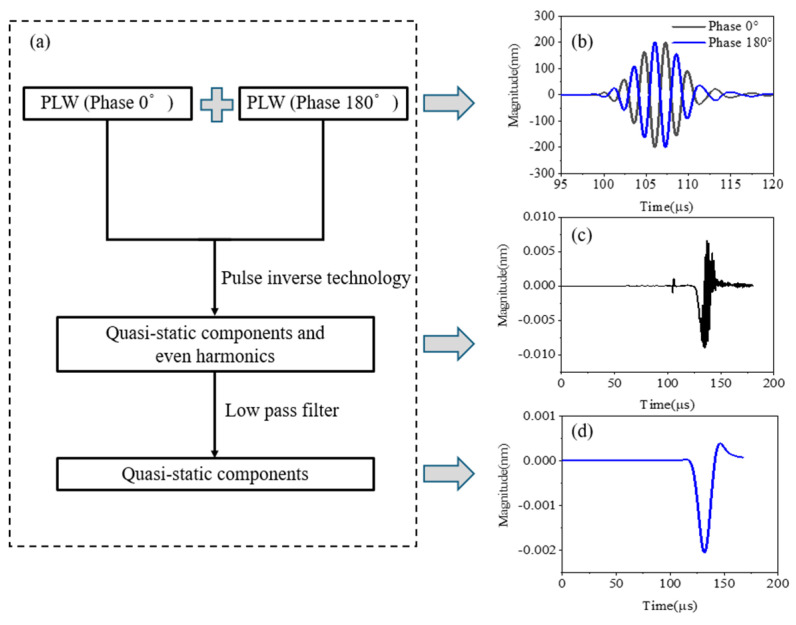
The extraction process of QSC: (**a**) flow chart, (**b**) sum of PLW with phase difference of 180°, (**c**) QSCs and even order harmonics, and (**d**) QSC pulse.

As shown in Figure 7a,b, two excitation signals with a phase difference of 180° can be added after propagating in nonlinear media to cancel the PLW and the generated odd harmonics; that is, the pulse reversion technique [42], which would suppress the even harmonic components and magnified the odd ones, can obtain the superposition signal of the QSC and even harmonics, as shown in Figure 7c. Since the frequency of the QSC can be nearly regarded as 0 MHz, the pure QSC, as shown in Figure 7d, can be obtained by the 0.2 MHz cut-off frequency low-pass filtering processing.

The processing procedure for obtaining QSC directivity is as follows:

Step 1: Extract the corresponding time-domain signal from each monitoring point;

Step 2: Extract pure QSC signals by using the method shown in Section 2.5;

Step 3: Extract the peak value of the QSC corresponding to the monitoring point;

Step 4: Convert the peak value of the QSC of each monitoring point from the Cartesian coordinate system to the polar coordinate system;

Step 5: Obtain micro-crack direction diagram of the QSC.

In order to analyze the effect of the micro-crack orientation on QSC directivity, a micro-crack is numerically simulated at different orientation angles.

## 3. Simulation Results and Discussions

**Figure 8:**
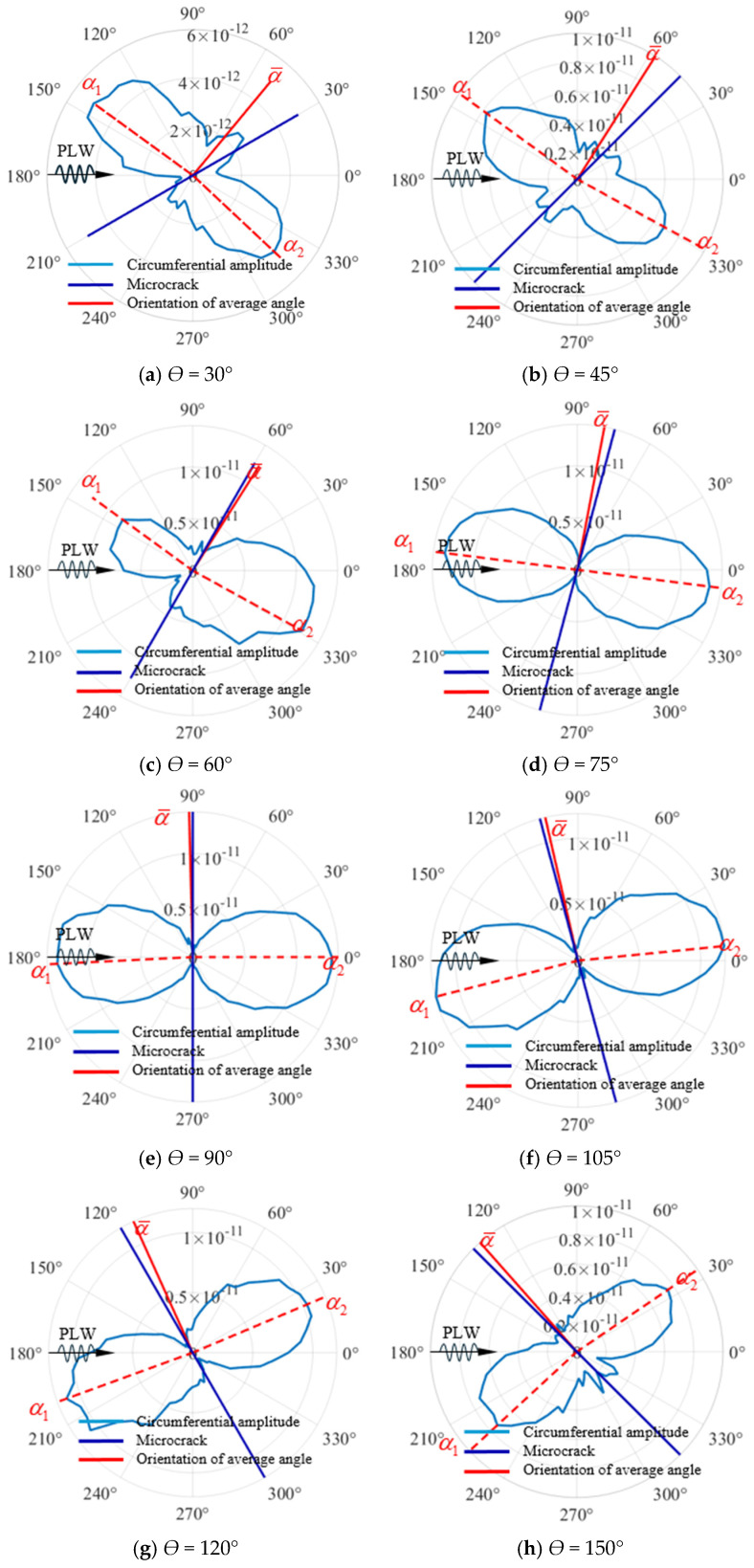
QSC directivity under different micro-crack angles.

When the relevant parameters of the incident PLW and micro-crack are given, that is, after the parameters (such as $A_{inc},f_{inc},\alpha$) in Equation (2) are given, the relevant directivity characteristics of the QSC sound field $U_{0f}$ generated by the micro-crack are closely related to the orientation angle of the micro-crack α. Therefore, the directivity of the QSC generated by the micro-crack can be used to evaluate quantitatively. To study the quantitative relationship between them, the micro-crack models of the same size but different orientations are simulated in FEM. The orientation angle α of the micro-crack ranges from 30° to 150° with an increment of 15°, and the directivity of the QSC sound field and the examined micro-crack orientation is quantitatively analyzed. The monitoring point is located around the micro-crack with a circle radius of 20 mm (see Figure 4), and the horizontal displacement component is recorded. The directivity diagram of the QSC in the polar coordinate system is shown in Figure 8a–h. It can be seen that the directivity of the QSC varies sensitively with the change in the micro-crack orientation, as shown in Figure 8. By observing the change rule of the directivity of the QSC with the orientation of the micro-crack, the average magnitude value of the maximum one distributed on both sides of the micro-crack is defined as the average angle $\bar{\alpha }=\left(\alpha_{1}+\alpha_{2}\right)/2$. It can be concluded that the average angle is approximately equal to the orientation angle of the micro-crack. Therefore, the average angle $\bar{\alpha }$ can be used as the benchmark for evaluating the orientation angle of the examined micro-crack. In conclusion, the directivity of the QSC can be used for determining the characteristic orientation of the micro-cracks.

It is found that the directivity of the QSC generated by the micro-crack is sensitive to the change in the angle of the micro-crack, and the defined average angle is in good agreement with the position of the micro-crack. Based on these characteristics, the direction of the micro-crack can be characterized by QSC directivity.

**Figure 9:**
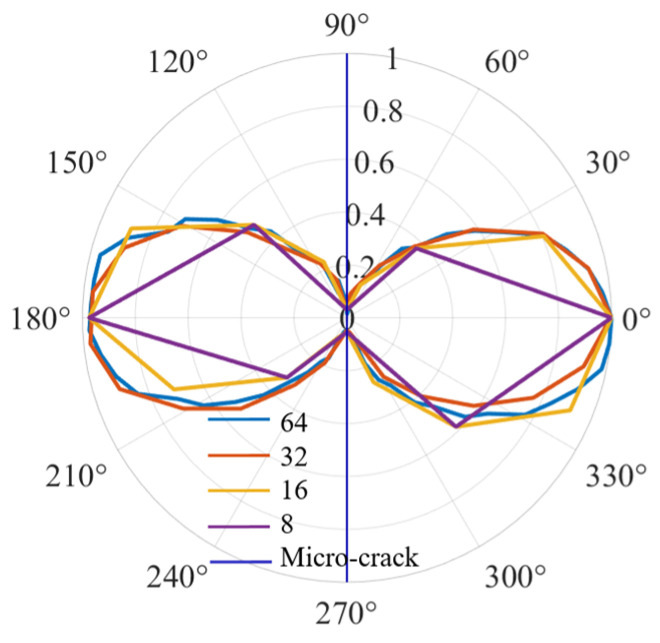
QSC directivities with different monitoring points number.

To accurately evaluate the micro-crack orientation, the monitoring number is set to 64 in Figure 8, so that the curves of the QSC directivity patterns are smooth. In fact, there are not so many monitoring points in the experiments. Therefore, different monitoring point numbers for characterizing QSC directivity are discussed, as shown in Figure 9. It can be seen that the normalized curves of the QSC directivities become zigzag with the reduction in the number of monitoring points. But even if the monitoring points number is set to be eight, the micro-crack orientation can be characterized by QSC directivity.

**Figure 10:**
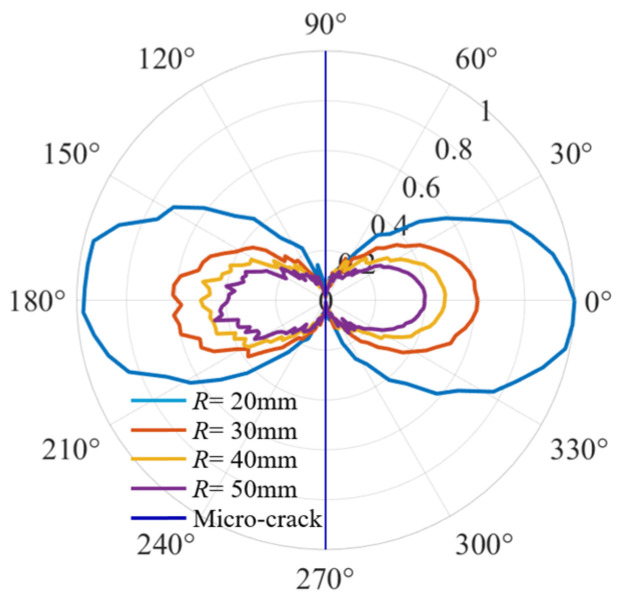
QSC directivities under different radii.

It can be seen that the normalized curves of QSC directivity become more and more zigzag with the increase in the distribution radii of the monitoring points, as shown in Figure 10. At the same time, the amplitudes decrease gradually. However, the orientation of micro-cracks remains unchanged regardless of variations in the distribution radii of the monitoring points.

**Figure 11:**
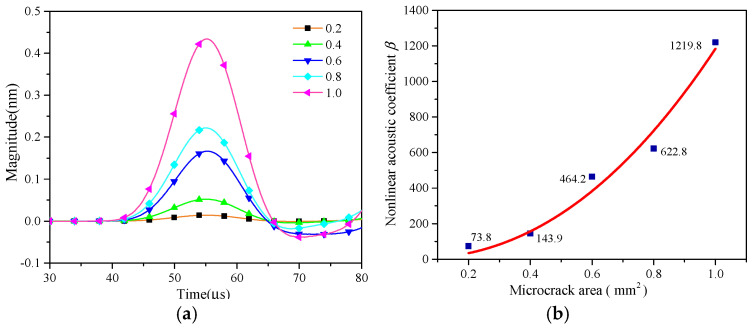
(**a**) Time-domain signal of QSC with different areas of micro-crack, and (**b**) relative acoustic nonlinear parameter at a monitoring point (423.6, 1.0) mm.

It is worth mentioning that the amplitude of the QSC is affected by the change in the elasticity modulus ΔC(t). The acoustic nonlinearity parameter $\left(A_{0}/A_{1}\right)$ is used for intuitively exhibiting the relationship between the local stiffness change and the amplitude of the QSC. The time-domain signals and relative acoustic nonlinearity parameters versus the stiffness difference are shown in Figure 11a,b, respectively. When the contact area increases from 0.2 to 1.0 mm^2^, the amplitude of the QSC changes and monotonically increases, as shown in Figure 11a, and the relative acoustic nonlinearity parameter increases linearly with the contact area change, as shown in Figure 11b.

## 4. Conclusions

This paper aims to quantitatively characterize the orientation of the micro-crack. QSC directivity is related to the micro-crack orientation. It is worth noting that PLWs propagating in weak nonlinear materials will produce the QSC. In order to avoid the nonlinear influence from the materials, the group velocity of the PLW is mismatched with that of the generated QSC. In this point, the micro-crack can be deemed the only nonlinear source generating the QSC. To quantitatively evaluate the relationship between QSC directivity and the micro-crack orientation, the FEM is built. The simulation results show that the directivity of the QSC sensitivity changes with the variation in micro-crack orientation. A mean angle based on the QSC sound field is defined for evaluating the micro-crack orientation, which is in good agreement with its theoretical value, and it can be characterized by this angle without a baseline signal.

## Figures and Tables

**Table 1:** Material parameters of the aluminum used in FE simulations.

Density (kg/m^3^)	Elasticity Modulus (GPa)	Poisson’s Ratio	Group Velocity of PLW (m/s)	Group Velocity of QSC (m/s)
2700.0	69.0	0.33	3067.4	5345.4

## Data Availability

The original contributions presented in this study are included in the article material. Further inquiries can be directed to the corresponding author.
